# Supplementary material for: Causal Relationship between Aging and Anorexia Nervosa: A White-Matter-Microstructure-Mediated Mendelian Randomization Analysis
Source: Biomedicines. 2024 Aug 16;12(8):1874. doi: 10.3390/biomedicines12081874 (PMC11351342; doi:10.3390/biomedicines12081874)
Supplement: Supplementary file 1 [file biomedicines-12-01874-s001.zip › Supplementary Figure/Figure S4/Singe SNP plot of MR analysis for TL to ubm-b-1924.pdf]

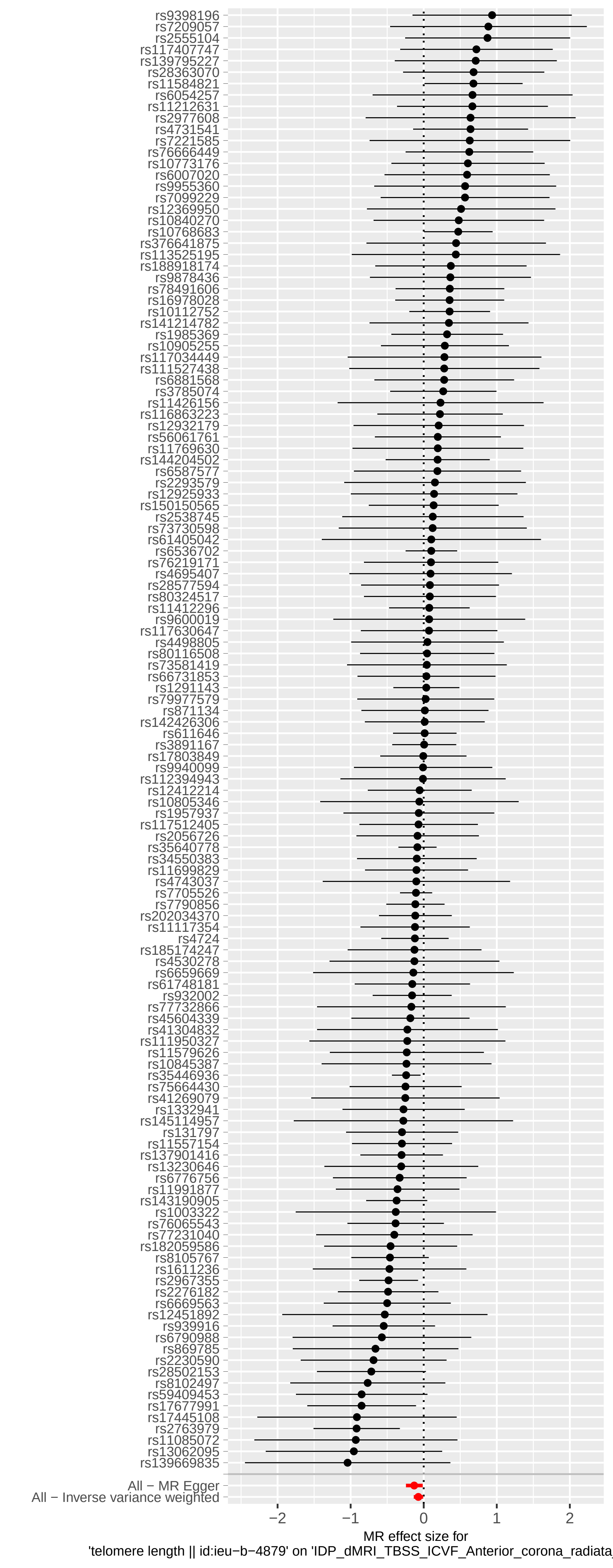
rs9398196
rs7209057
rs2555104
rs117407747
rs139795227
rs28363070
rs11584821
rs6054257
rs11212631
rs2977608
rs4731541
rs7221585
rs76666449
rs10773176
rs6007020
rs9955360
rs7099229
rs12369950
rs10840270
rs10768683
rs376641875
rs113525195
rs188918174
rs9878436
rs78491606
rs16978028
rs10112752
rs141214782
rs1985369
rs10905255
rs117034449
rs111527438
rs6881568
rs3785074
rs11426156
rs116863223
rs12932179
rs56061761
rs11769630
rs144204502
rs6587577
rs2293579
rs12925933
rs150150565
rs2538745
rs73730598
rs61405042
rs6536702
rs76219171
rs4695407
rs28577594
rs80324517
rs11412296
rs9600019
rs117630647
rs4498805
rs80116508
rs73581419
rs66731853
rs1291143
rs79977579
rs871134
rs142426306
rs611646
rs3891167
rs17803849
rs9940099
rs112394943
rs12412214
rs10805346
rs1957937
rs117512405
rs2056726
rs35640778
rs34550383
rs11699829
rs4743037
rs7705526
rs7790856
rs202034370
rs11117354
rs4724
rs185174247
rs4530278
rs6659669
rs61748181
rs932002
rs77732866
rs45604339
rs41304832
rs111950327
rs11579626
rs10845387
rs35446936
rs75664430
rs41269079
rs1332941
rs145114957
rs131797
rs11557154
rs137901416
rs13230646
rs6776756
rs11991877
rs143190905
rs1003322
rs76065543
rs77231040
rs182059586
rs8105767
rs1611236
rs2967355
rs2276182
rs6669563
rs12451892
rs939916
rs6790988
rs869785
rs2230590
rs28502153
rs8102497
rs59409453
rs17677991
rs17445108
rs2763979
rs11085072
rs13062095
rs139669835
All − MR Egger
All − Inverse variance weighted
−2
−1
0
1
2
MR effect size for
'telomere length || id:ieu−b−4879' on 'IDP_dMRI_TBSS_ICVF_Anterior_corona_radiata_
